# Development of a Rhizobium Seed Coating to Establish Lupine Species on Degraded Rangelands

**DOI:** 10.3390/plants13152101

**Published:** 2024-07-29

**Authors:** Bridget M. Church, Brad Geary, Joel Griffitts, Curtis L. Drake, Kate Ruebelmann, Shannon V. Nelson, Matthew D. Madsen

**Affiliations:** 1 Department of Plant and Wildlife Sciences, Brigham Young University, Provo, UT 84602, USA; calderbridget@gmail.com (B.M.C.); brad_geary@byu.edu (B.G.); curtisdrake10@gmail.com (C.L.D.); nelson.shannonvalerie@gmail.com (S.V.N.); 2 Department of Microbiology and Molecular Biology, Brigham Young University, Provo, UT 84602, USA; joelg@byu.edu; 3 Rehabilitation, Projects & Engineering, Rio Tinto Kennecott, South Jordan, UT 84095, USA; kate.ruebelmann@riotinto.com

**Keywords:** biochar, compost, inoculum, microorganisms, *Lupinus*, restoration

## Abstract

Restoring native plant species on degraded landscapes is challenging. Symbiotic partners in the plant rhizosphere can aid in nutrient acquisition, pathogen protection, stress tolerance, and many other processes. However, these microbes are often absent in altered landscapes and need to be re-integrated to improve restoration efforts. We evaluated, within a laboratory setting, the ability of commercial and indigenous rhizobia strains to form nodules on lupine species used for rangeland seedings in the Great Basin region of the Western United States and ascertained if these strains could be applied through a seed coating. We also evaluated if a compost amendment applied via seed coating could further enhance the performance of the rhizobia strains. Our analysis showed that successful nodulation could occur using commercial and wildland-collected indigenous strains through either a liquid culture applied to seedlings or as a dry seed coating. However, the number of root nodules and the presence of a pink color (indicating nitrogen fixation) were typically higher in the commercial product than in the indigenous strains. Compost did not improve nodulation or the performance of the nodules; however, this treatment alone improved shoot growth. Overall, these results suggest that commercial rhizobium may be more effective in improving plant growth, and future research with native rhizobia may want to consider identifying strains compatible with seed-coating delivery. Longer-term studies are now merited for assessing how the rhizobia strains evaluated in this study influence plant growth, particularly in a field setting.

## 1. Introduction

Organisms of all kinds interact daily in ways we may not realize [1]. These symbiotic interactions can be necessary for survival and are especially important for sessile organisms such as plants [2]. Plant symbiotic partners may reside in the rhizosphere or directly within the tissues as endophytes. These microorganisms, such as mycorrhizal fungi and nitrogen-fixing bacteria, can help with pathogen protection, stress tolerance [3,4,5], and nutrient acquisition [6,7]. Because these microorganisms offer so many benefits, their presence in the soil can be an important contributing factor to the successful establishment of native plant species [8,9,10].

Most often, these microorganisms are not present in areas with degraded soils or altered landscapes because they rely on the presence of their plant host species [11,12,13]. Landscape alterations may come from overgrazing, soil tillage, chemical fertilizers and pesticides, mineral extraction, and urban expansion [14,15,16]. A necessary step in restoring native landscapes is re-integrating these symbiotic microorganisms into the soil with the plants [17,18].

The reintroduction of nitrogen-fixing bacteria (rhizobia), in combination with their associated legume host plant, can affect successional trajectories by facilitating colonization and growth of subsequent species that are limited by soil organic matter and the availability of critical nutrients such as nitrogen [19,20,21]. Nitrogen-fixing bacteria provide the greatest source of fixed nitrogen to terrestrial environments [22]. When near their specified partner, rhizobia induce nodulation of the legume roots. In the nodule, the rhizobia fix atmospheric nitrogen into ammonium, and in return, the plant provides the necessary sugars to feed the bacteria [23]. This coalescence of metabolisms allows both partners to thrive in less-than-optimal conditions [13,24,25]. 

Seed-coating technologies that deliver rhizobia at the time of planting may be especially useful in improving restoration efforts in severely degraded landscapes [14,26]. Seed coating optimizes the delivery of bacteria by placing them in proximity to the host plant [27,28,29]. Despite the potential benefits that rhizobia-coated seeds could provide to restoration efforts, minimal research has been directed toward optimizing this technology for this purpose. Restoration efforts could benefit from research that identifies rhizobia strains that are compatible with the host plant being sown and the site being restored.

When the host plant and the rhizobia lack compatibility, it can result in either a lack of nodulation or nodulation occurring without subsequent nitrogen fixation [30,31]. Additionally, even when the rhizobia are compatible, some strains may perform better with respect to such processes as nodule formation and nitrogen fixation [32,33,34]. Because indigenous strains are adapted to their native soils and environment, they may perform better than non-native strains [34]. Alternatively, commercial strains of rhizobia are easier to obtain and are available for most legume restoration species. They are often selected for their ability to produce nitrogen and be delivered through a seed coating [23]. However, there is minimal research showing how these strains will perform outside of an agricultural system. Additionally, in many systems, sufficient research has not been performed to understand if a commercial rhizobia inoculum is adequate or if indigenous rhizobia strains need to be collected and cultured to meet restoration goals. 

Research is also lacking in wildland systems on methods for delivering rhizobia through a seed coating. Unlike in agriculture, where the seeds are sown during a period that is conducive to germination, seeds sown in wildland systems may sit dormant in harsh conditions for several months before they germinate [35]. Seed-coating methods and materials that maximize the rhizobia’s survival over the dormant season could aid in the success of this treatment in ecological restoration projects. 

Ground and sterilized peat is a favorable medium for the growth of bacteria and is a useful carrier for the organisms within a seed coating [36,37,38]. More recently, biochar has been shown to be a suitable inoculant carrier, which is thought to be due to its ability to protect microorganisms, hold moisture, and provide a potential source of energy and minerals [39]. Compost has also been shown to have a synergistic effect when combined with plant-associated microbes, such as rhizobia [40]. The increased rhizobium activity produced by the compost may be due to the material acting as a carbon and nutrient source [41]. Additionally, including compost in a seed coating may further aid in the success of a restoration project by supplying the seed with a diversity of beneficial microbes [42] that can suppress plant diseases [43] and aid in plant growth [44]. 

This research was conducted on silky (*Lupinus sericeus* Pursh) and silvery lupine (*Lupinus argenteus* Pursh). Lupine species, in general, are useful in restoring severely degraded rangelands, as their symbionts help increase soil nitrogen levels, promoting the growth of other native species and advancing successional processes [21,45]. Silky and silvery lupine are favored for their bright purple flowers and ability to add biodiversity, benefit pollinators, and provide forage for wildlife [46]. 

The objectives of our research were to (1) determine if indigenous rhizobia strains induce nodulation of native lupine species and compare their performance against a commercial rhizobium inoculant; (2) ascertain if these strains could be applied through a seed coating while maintaining bacterial viability; and (3) determine if incorporating a blend of biochar and compost into the coating process will aid in the effectiveness of rhizobia coatings. We hypothesized that (1) commercial and indigenous rhizobia strains would successfully nodulate the roots of lupine when applied directly to an immature seedling or when added to a seed coating; (2) the performance of the rhizobia strains would vary in regard to their ability to promote plant growth; and (3) the application of a blend of compost and biochar in conjunction with a rhizobia coating would further enhance seedling establishment and plant growth. 

This article presents research on an understudied topic, with the potential to significantly bolster restoration efforts for lupine and legume species in general. It also provides valuable insights that could guide future laboratory and field studies aimed at improving methods for applying rhizobium seed coatings to restore native plant communities. Our research was conducted in three separate laboratory trials. The first study (hereafter referred to as EXCEED^®^ Trial) was implemented to test the efficacy of the commercial rhizobium product EXCEED^®^ H Type Inoculant for Lupine (Visjon Biologics, Henrietta, TX, USA) in its ability to improve plant growth when delivered through a seed coating. The next two trials focused on understanding the performance of four indigenous rhizobia strains in comparison to each other and that of the commercial strain tested in the EXCEED^®^ trial. The indigenous rhizobium used in this study was harvested from lupine species at Rio Tinto Kennecott’s Bingham Canyon Mine, Herriman, UT, USA (40.501556, −112.141833); Lupine Hiking Trail, Draper, UT, USA (40.464417, −111.828694); and near Strawberry Reservoir, Heber, UT, USA (40.15440, −111.20236). In the first indigenous rhizobia strain trial, we tested the inoculants when they were delivered as a liquid culture onto pre-germinated seeds (hereafter referred to as the Inoculation Trial). This study allowed us to evaluate the rhizobium treatments’ performance without the interaction of the seed coating. In the second indigenous rhizobia strain trial, we evaluated the inoculants when applied through a seed coating (hereafter referred to as the Coating Trial). Additionally, within this seed-coating trial, we assessed whether adding a compost amendment within the seed coating would enhance the rhizobia’s performance and the plant’s subsequent growth. 

## 2. Results

### 2.1. EXCEED^®^ Trial: Evaluation of a Commercial Rhizobia Product

**Figure 1:**
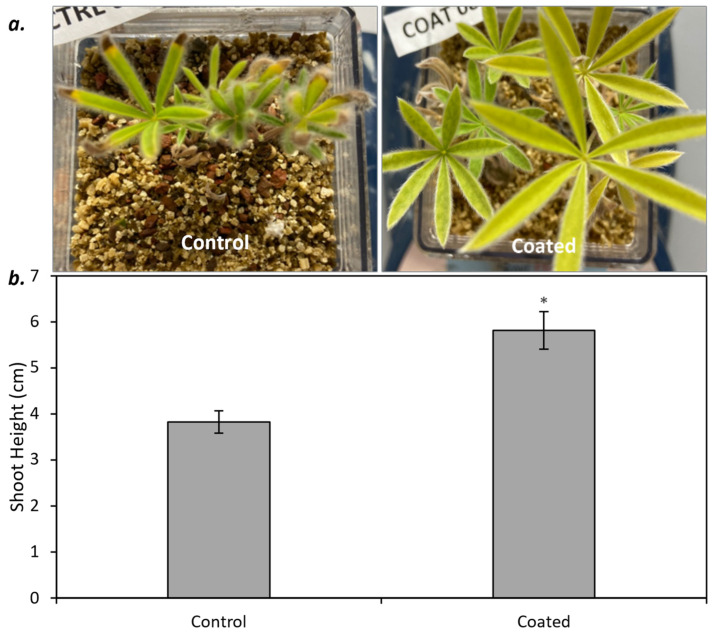
Photos (**a**) and seedling shoot height (±SE) (**b**) for silky lupine (*Lupinus sericeus* Pursh) grown from control (uncoated) and rhizobia-coated seeds. Asterisks represent significance (*p* < 0.05).

There was no difference in seedling emergence between the control (uncoated) and coated seed, with emergence at 44.4 ± 8.1% and 50.0 ± 5.1%, respectively (*p* = 0.075; *t* = 1.92). Plants grown from coated seeds had a 53% increase in shoot height relative to the control (*p* = 0.008; *t* = 3.09; Figure 1). While harvesting the study, we observed that the plants grown from coated seed had nodules. In contrast, plants from untreated seed generally lacked nodules and were relatively smaller in size when present compared to the seed-coating treatment. An analysis of plant tissue with all replicates combined showed that plants grown from coated seed had a 47% increase in shoot nitrogen content (control = 14.4 g kg^−1^, coated = 21.2 g kg^−1^) and 40% increase in root nitrogen content (total N for the control = 11.1 g kg^−1^; coated = 15.5 g kg^−1^), respectively, relative to the control.

### 2.2. Inoculation Trial: Evaluation of Indigenous Rhizobia Strains on Root Nodulation and Plant Growth When Applied as a Liquid Culture to Pre-Germinated Seeds

**Figure 2:**
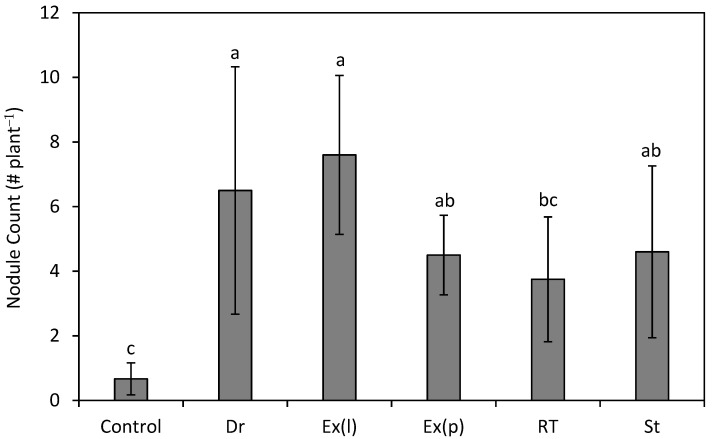
Mean (±SE) number of root nodules on silvery lupine (*Lupinus argenteus* Pursh) seedlings inoculated with liquid cultures isolated from Draper, UT (Dr); Rio Tinto Kennecott Copper Mine, Herriman, UT (RT); Strawberry Reservoir, Heber, UT (St); and EXCEED^®^ liquid (Ex(l)) and peat-based (Ex(p)) commercial inoculum. Different lowercase letters indicate a significant difference between treatments (*p* < 0.05).

**Figure 3:**
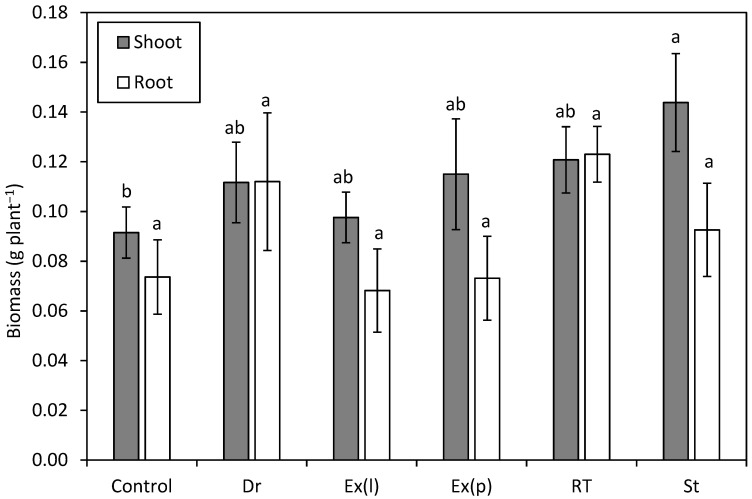
Mean (±SE) shoot and root dry weight (g plant^−1^) of silvery lupine seedlings inoculated with liquid cultures of rhizobium isolated from plants collected near Draper, UT (Dr); Rio Tinto Kennecott Copper Mine, Herriman, UT (RT); and Strawberry Reservoir, Heber, UT (St), as well as from the commercial inoculum EXCEED^®^ delivered to the seed as either a liquid inoculum (Ex(l)) or a peat based inoculum (Ex(p)). Different lowercase letters indicate a significant difference between treatments (*p* < 0.05).

Rhizobia strains from Draper (Dr); Strawberry Reservoir (St); and the commercial inoculums, Exceed^®^ liquid (Ex(l)) and Exceed^®^ peat (Ex(p)), had more root nodules in comparison to the control (*p* < 0.05; Figure 2). While higher on average, the number of nodules produced from plants grown with Rio Tinto (RT) inoculum was not significant from the control but trended in that direction (*p* = 0.050; Figure 2). There was no difference between the inoculation treatments for shoot height (*p* = 0.566) or root weight (*p* = 0.066), but there was a difference in shoot weight (*p* = 0.017). Here, the St isolate produced shoots that were 57% larger than the control (*p* = 0.031), but no other treatments were significant for this metric (Figure 3). An analysis of plant tissue with all replicates combined showed that the plants grown from germinated seeds inoculated with the Dr and Ex(l) rhizobia strains had a 31% and 40% increase in total shoot nitrogen, respectively, compared to the control (total N of the control = 19.9 g kg^−1^, Dr = 26.1 g kg^−1^, and Ex(l) = 27.9 g kg^−1^). The Ex(l) and Ex(p) rhizobia treatments increased the total root nitrogen relative to the control by 38 and 110% (total N of the control = 11.9 g kg^−1^, Ex(l) = 16.4 g kg^−1^, and Ex(p) = 25.0 g kg^−1^), respectively.

### 2.3. Coating Trial: Evaluation of Indigenous Rhizobia Strains Applied with Different Seed Coatings

**Figure 4:**
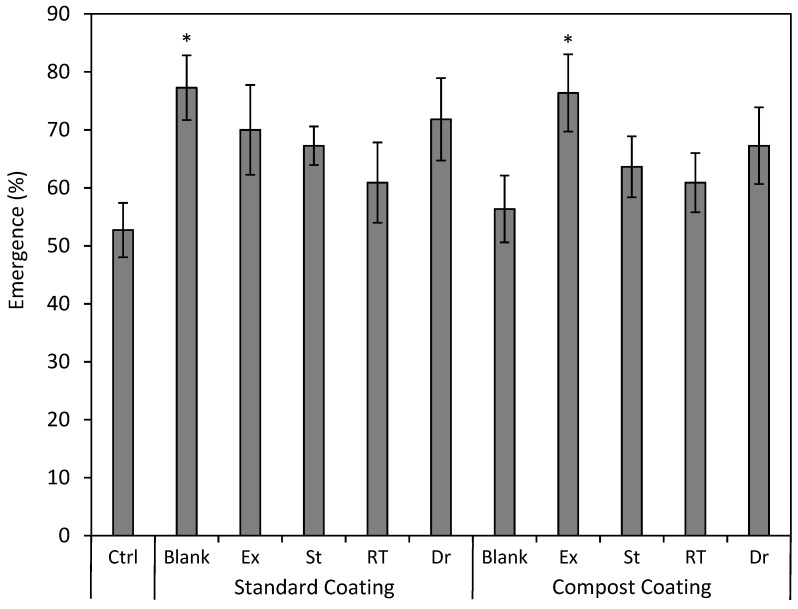
Mean (±SE) seedling emergence (%) of silvery lupine (*Lupinus argenteus* Pursh) from seeds coated with the commercial rhizobium strain EXCEED^®^ (Ex) and rhizobium strains collected near Strawberry Reservoir, Heber, UT (St); Rio Tinto Kennecott Copper Mine, Herriman, UT (RT); Draper, UT (Dr). The rhizobium inoculant was incorporated in either the standard seed coating (wood flour, powdered cellulose, calcium bentonite, and peat moss) or the compost seed coating (compost and peat moss). Asterisks represent significant differences from the control (*p* < 0.05).

**Figure 5:**
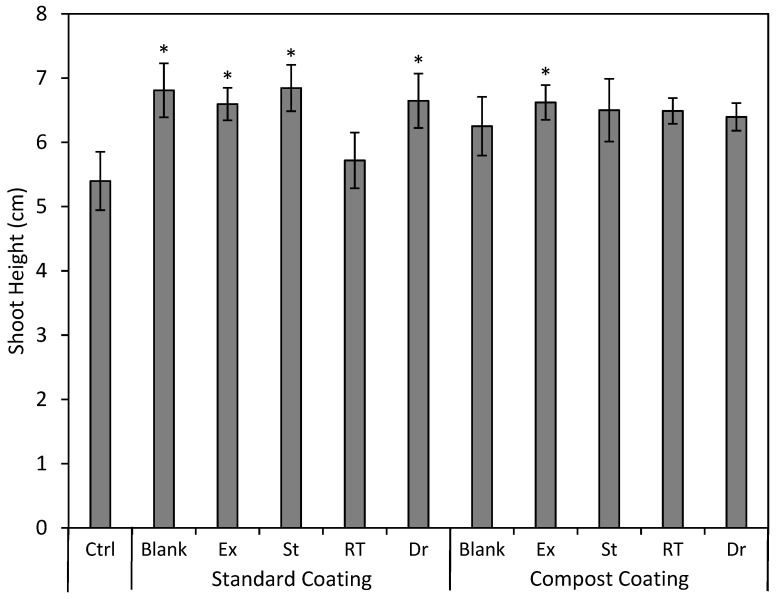
Mean (±SE) shoot height (cm) of silvery lupine (*Lupinus argenteus* Pursh) from seeds coated with the commercial rhizobium strain EXCEED^®^ (Ex) and rhizobium strains collected near Strawberry Reservoir, Heber, UT (St); Rio Tinto Kennecott Copper Mine, Herriman, UT (RT); and Draper, UT (Dr). The rhizobium inoculant was incorporated in either the standard seed coating (wood flour, powdered cellulose, calcium bentonite, and peat moss) or the compost seed coating (compost and peat moss). Asterisks represent significant differences from the control (*p* < 0.05).

The standard coating with no added bacteria (SC Blank) and the compost coating + Exceed^®^ (CC + Ex) were the only two treatments that showed a statistical improvement in emergence, with a 24.5% (*p* = 0.039) and 23.6% (*p* = 0.029) increase over the control, respectively (Figure 4). All the treatments had an overall effect on shoot height (*p* = 0.025). The Tukey analysis showed no difference between any two treatments, but Dunnett’s test showed increased shoot height over the control for SC Blank, CC + EXCEED^®^, SC + St, SC + Dr, and SC + EXCEED^®^, with lengths ranging between 22 and 27% higher than the control (*p* = 0.014–0.046; Figure 5). 

**Figure 6:**
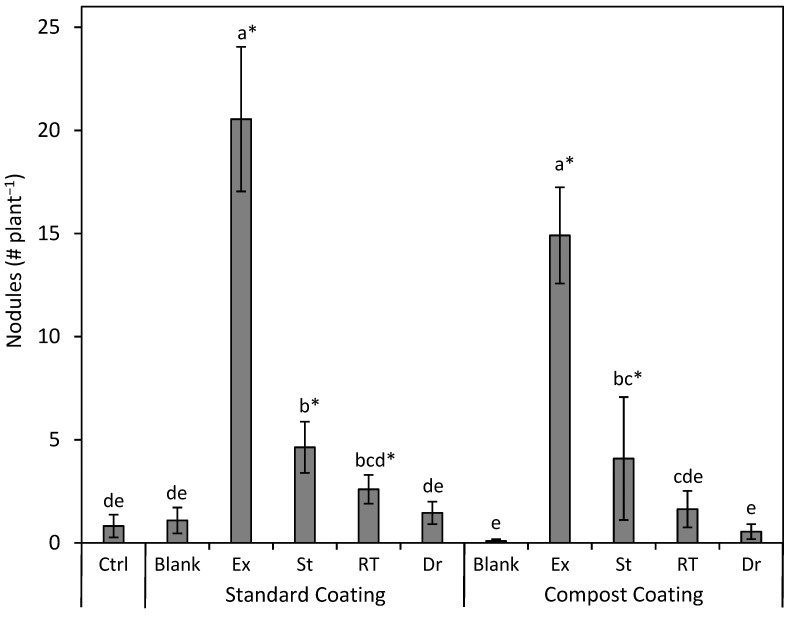
Mean (±SE) nodule count (# plant^−1^) on silvery lupine (*Lupinus argenteus* Pursh) roots from seeds coated with the commercial rhizobium strain EXCEED^®^ (Ex) and rhizobium strains collected near Strawberry Reservoir, Heber, UT (St); Rio Tinto Kennecott Copper Mine, Herriman, UT (RT); and Draper, UT (Dr). The rhizobium inoculant was incorporated in either the standard seed coating (wood flour, powdered cellulose, calcium bentonite, and peat moss) or the compost seed coating (compost and peat moss). Different lowercase letters indicate a significant difference between all treatments (*p* < 0.05). Asterisks represent a significant difference in the individual treatment compared only to the control (*p* < 0.05).

There was a drastic difference in the number of root nodules produced by the different seed-coating treatments (*p* < 0.001; Figure 6). The SC and CC coatings with EXCEED^®^ were the top-performing treatments, with EXCEED^®^ having between 4.4 and 14.1-fold and between 3.6 and 27.3-fold more nodules than the indigenous bacteria in the SC and CC coating groups, respectively. In addition to EXCEED^®^ coatings, nodule counts were higher than the control for SC + St (*p* < 0.0001), SC + RT (*p* = 0.0207), and CC + St (*p* = 0.0002). The analysis of shoot tissue with all replicates combined showed a minimal increase in total nitrogen relative to the control for the SC + Ex and CC + Ex treatments (total N of the control = 22.6 g kg^−1^, SC + Ex = 22.9 g kg^−1^, and CC + Ex = 2.25 kg^−1^). All other treatments had total shoot nitrogen contents that were slightly less than the control. Root nitrogen from SC + Ex and CC + Ex treatments was 50% and 39% higher than the control, respectively (total N of SC + Ex = 19.3 g kg^−1^, CC + Ex = 18.0 g kg^−1^). Also notable is that CC + St was 65% higher than the control (total N of CC + St = 2.13 g kg^−1^). The remainder of the rhizobia treatments also showed total root nitrogen levels numerically larger than the control, ranging from 3.9 to 23% more than the control. 

**Figure 7:**
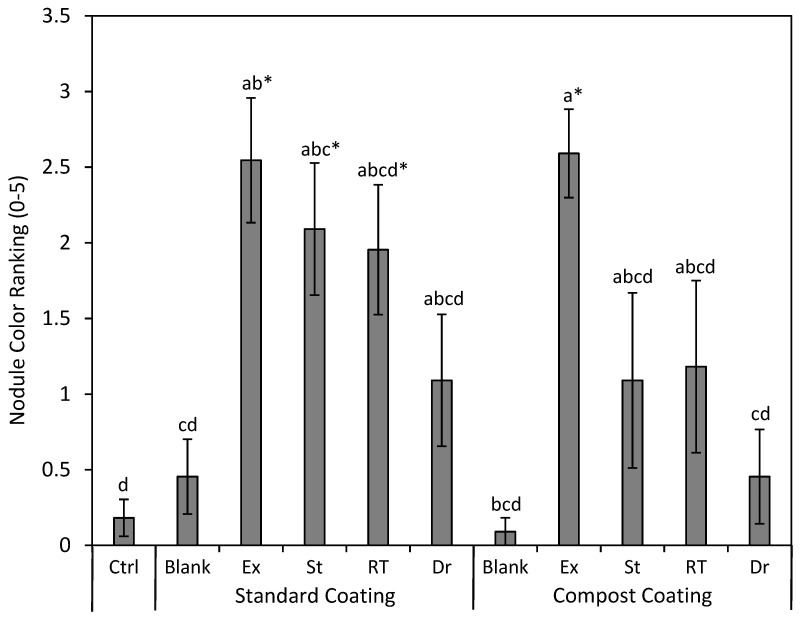
Mean (±SE) ranking of nodule color of silvery lupine (*Lupinus argenteus* Pursh) from seeds coated with the commercial rhizobium strain EXCEED^®^ (Ex) and rhizobium strains collected near Strawberry Reservoir, Heber, UT (St); Rio Tinto Kennecott Copper Mine, Herriman, UT (RT); and Draper, UT (Dr). The rhizobium inoculant was incorporated in either the standard seed coating (wood flour, powdered cellulose, calcium bentonite, and peat moss) or the compost seed coating (compost and peat moss). Plants with no nodules were given a rank of zero; if the nodules were white, brown, or green they were given a value of 1; and plants with a pink/red color were given a score of 2–5 depending on the darkness of the nodules. Different lowercase letters indicate a significant difference between all treatments (*p* < 0.05). Asterisks represent a significant difference in the individual treatment compared only to the control (*p* < 0.05).

In general, all the treatments with rhizobia strains had pink nodules, indicating that active nitrogen fixation was occurring, while the few nodules growing on plants in the control or coatings without rhizobia were lacking in this color (Figure 7; *p* < 0.001). The degree of pink in the nodules was highest on average for EXCEED^®^; however, statistically, most other rhizobia treatments had a similar color to this product. However, when analyzed with a Dunnett’s test, only SC + EXCEED^®^, SC + St, SC + RT, and CC + EXCEED^®^ had a higher pink rating than the control (*p* = 0.002–0.008; Figure 7).

**Figure 8:**
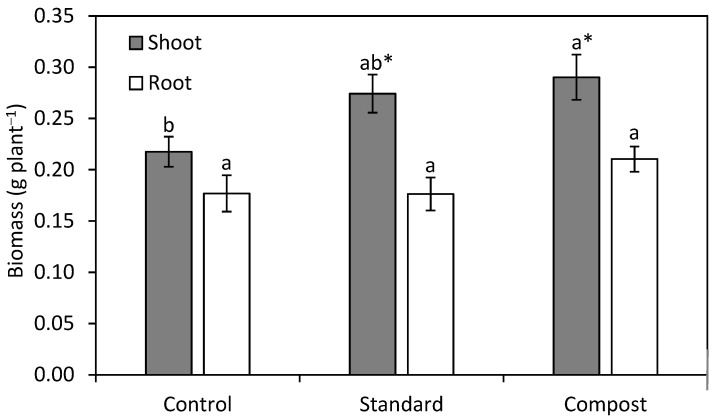
Mean (±SE) shoot and root weight among different coatings on silvery lupine (*Lupinus argenteus* Pursh). Seeds were either left uncoated or coated with a standard coating or a compost coating. Different lowercase letters indicate a significant difference between all treatments (*p* < 0.05). Asterisks represent a significant difference in the individual treatment compared only to the control (*p* < 0.05).

Lastly, no overall treatment effect was detected for shoot and root biomass (shoots *p* = 0.067; roots *p* = 0.183). However, when the rhizobia treatments were excluded from the analysis, there was a significant effect on shoot biomass (*p* = 0.025) but not root biomass (*p* = 0.192). The CC treatment had a 33% increase in shoot weight in comparison to the control (*p* = 0.026), while the SC showed an intermediate response between the control and the CC treatment (Figure 8).

## 3. Discussion

The establishment and long-term survival of native plant species on degraded and altered landscapes can be a major challenge to restoration efforts. One major impediment can be the absence of vital soil symbionts that allow for species’ success in low-nutrient areas [7]. By incorporating microorganisms directly into a seed coating, restoration practitioners can better ensure site-specific inoculation into the plant rhizosphere [14]. 

As predicted by our hypotheses, we found in the EXCEED^®^ Trial that applying a commercially produced inoculum through a seed coating induced root nodulation and improved plant growth. These results demonstrate that a rhizobium seed coating can enhance plant growth in media with limited nitrogen availability. We attribute this improvement in plant growth to the symbiotic relationship between rhizobium bacteria and the host plant, which facilitates nitrogen fixation, thereby providing essential nutrients that promote healthy development [7,32,33,34]. The implications of these findings are substantial for the restoration of degraded systems such as rangelands and minelands. By enhancing the establishment and growth of native plant species in nitrogen-poor soils, rhizobium seed coatings may accelerate the recovery of these ecosystems, improve soil health, and support biodiversity. This innovative approach offers a promising solution to the challenges faced in wildland restoration efforts, contributing to more sustainable and resilient landscapes. Based on these promising laboratory results with the commercial product EXCEED^®^, future research is merited to prove the effectiveness of this seed coating in the field.

Our Inoculation Trial, where we applied inoculum to pre-germinated seeds via a liquid culture, allowed us to evaluate the performance of commercial and indigenous strains when applied without a seed coating. The study results demonstrate that similar nodulation can generally be achieved between commercial inoculum and wildland-collected indigenous strains. In this study, there was an increase in the number of nodules over the control from all the treatments except for the RT strain, which was not quite significantly different from the control (*p* = 0.0503; Figure 2). However, in our Coating Trial, when the different rhizobia treatments were applied through a seed coating, the commercial inoculum far surpassed the indigenous strains in the number of root nodules produced (Figure 6). These results highlight the need to select compatible rhizobia strains for the species being inoculated and indicate that it may also be important to screen for rhizobia strains that perform well when applied as a seed coating. It can be assumed that commercial rhizobia products are developed through rigorous selection and optimization by companies, selecting strains that demonstrate high efficiency in nodulation, nitrogen fixation, and overall plant growth under various conditions [23]. Future efforts to find locally sourced rhizobia may want to consider screening multiple populations to identify strains with high nodulation potential when applied through a seed coating.

It is also important that the rhizobia strains are effective in fixing nitrogen. In this study, the quality of the nodules, as assessed by ranking them by color, showed the indigenous rhizobia was statistically comparable to commercial inoculum (Figure 7). However, it may be important to note that the color ranking for the commercial inoculum was higher on average than the indigenous rhizobia strains. It is also interesting that the numerical comparisons of nitrogen content in the plant tissue were generally higher in the commercial rhizobia inoculums. These results lean towards suggesting that commercial rhizobium may be slightly more effective in delivering nitrogen to the plant. Thus, this study may further illustrate that future efforts to find locally sourced rhizobia may want to consider screening rhizobia based on their ability to increase plant nitrogen concentrations. 

We also saw some nodules form on treatments that did not have an inoculum. Nodules such as those could be due to other bacterial strains that cause nodulation but do not provide nitrogen fixation. Undesirable bacteria could have come from the seed, as well as the coating process. While seedlings were grown in a sterilized environment, the coating process was not sterile. Not only do these bacterial strains not fix nitrogen, but they also may inhibit the nodulation of rhizobia [9]. In addition, some rhizobia strains may nodulate and fix nitrogen but at such a low rate that the benefits are minimal. Not only are these strains poor nitrogen fixers, but they also may work to inhibit strains that would fix nitrogen more efficiently [47]. Future research in screening rhizobia strains for restoration efforts may want to evaluate how the strains compete against other strains that would naturally be on the seed or in the soil [47]. 

Our research from the Liquid Inoculation Trial provided modest evidence that a rhizobium inoculant would improve plant growth during the early stages of plant development. Here, we recorded increased shoot weight being produced from the St isolate, but while higher on average, the other rhizobium inoculant treatments were not statistically greater than the control. In our Coating Trial, no improvement in plant growth was recorded for any isolates when applied through a seed coating. Had this study been allowed to continue for longer, we may have seen a more considerable difference in plant growth. Mature nodules will fix more nitrogen than those found in immature root systems [48], increasing nitrogen production and plant growth. Additionally, the lack of response in our coated seeds could be due to the additional time it takes for the bacteria in the coating to proliferate, infect the plant’s roots, and cause nodulation. This would take longer than applying the liquid inoculum to pre-germinated seeds, as in the Inoculation Trial. Hence, these data collectively may indicate that future studies should run longer to better evaluate the rhizobia inoculum’s impact on plant growth. 

Our research indicated that the rhizobia inoculum had a minimal impact on seedling emergence. This lack of response in emergence suggests that the effects of rhizobia are not evident until more mature plant stages [48]. However, it is interesting to note that most of the coating treatments had higher emergence on average in comparison to the control, and for a couple of treatments, emergence was statistically higher (Figure 4). Improvements in emergence may be due to the seed-coating process conditioning the seed coat to allow for more rapid germination. 

Our final hypothesis was that the use of compost in conjunction with rhizobia would further enhance seedling growth and establishment. We found that compost did not improve nodulation or the performance of the nodules; however, this treatment alone improved shoot growth (Figure 8). This may be due to a higher level of nutrients present in compost that is not present in other coatings or our growing medium [44]. However, the minuscule amount of compost applied within the seed coating would provide minimal nutritional benefit to the plant. A more substantial advantage may be that the compost inoculates the growing medium with various microorganisms that can bring many benefits, such as increased nutrient mobilization, retention of water and nutrients, disease suppression, and improved soil function [11,49].

In conclusion, this study provides evidence that both commercial and indigenous strains of rhizobia can successfully nodulate the model lupine species tested in this study. However, for some metrics, the commercial product appears to outperform the indigenous strains. Future research is merited for furthering the development of the rhizobia coating for indigenous species. Establishing rhizobia back into the plant rhizosphere at the time of planting may help to provide a long-term solution to establishing species in poor soils. They may also work to establish other plant species by modifying soils back to pre-altered landscapes [19,20,21]. 

## 4. Materials and Methods

### 4.1. Collection, Isolation, and Preparation of Indigenous Rhizobia Strains

The roots of indigenous lupine plants were excavated from the soil and their nodules harvested at three different locations: Rio Tinto Kennecott’s Bingham Canyon Mine, Herriman, UT, USA (40.501556, −112.141833); Lupine Hiking Trail, Draper, UT, USA (40.464417, −111.828694); and near Strawberry Reservoir, Heber, UT, USA (40.15440, −111.20236). At each site, we obtained nodules from 5 to 10 individual plants. The Rio Tinto Kennecott site was a historic mining area now covered in waste rock, comprising weathered monzonite, quartzite, and limestone rock [50,51]. Its elevation is approximately 2000 m. Due to the extended time the waste rock has been deposited on site, it has been colonized by a small group of species, one of which is tailcup lupine (*Lupinus caudatus* Kellogg), from which we collected root nodules. The Lupine Hiking Trail is a Mountain Loam (Mountain Big Sagebrush) ecological site with a soil pH of 7.0 and a loam soil texture classification at an elevation of 1724 m [52]. Root nodules were collected from silvery lupine, one of the site’s dominant forbs. Our last collection site, near Strawberry Reservoir, is classified as a Mountain Loam ecological site. It is located at an elevation of 2430 m, with a loamy soil texture and pH of 7.2 [52]. This site contained multiple lupine species, and nodules were collected from silky, silvery, and bigleaf lupine (*Lupinus polyphyllus* Lindl.).

Upon collection, individual nodules were surface sterilized by swirling for 5 s in 75% ethanol, and then 25% bleach (5.25% sodium hypochlorite), followed by three consecutive dishes filled with autoclaved, distilled water (ddH_2_O). Following sterilization, nodules were crushed with a sterile pestle in an Eppendorf tube containing 100 uL tryptone yeast (TY) broth (Table 1). Once the bacteria had been released from the nodules, 5 uL of this liquid was spread onto a TY agar plate (Table 1) and placed in an incubator at 30 °C. 

Rhizobia strains were identified to the species level using 16s sequencing [53], transferred from the agar into 100 uL TY liquid broth solution, and incubated for 4 d at 30 °C. One rhizobia strain from each study site was selected for use in the study based on its ease of growing in liquid culture. Colony counts for each strain were determined from optical density (OD) measurements and plated liquid culture colony-forming unit (CFU) counts. Corresponding OD measurements were used to estimate CFU counts of our final liquid culture and calculate the correct dilution to match the commercial product’s (Exceed^®^ H Type Inoculant for Lupine (Visjon Biologics, Henrietta, TX, USA)) count of 2 × 10^9^ CFUs. 

### 4.2. EXCEED^®^ Trial: Evaluation of a Commercial Rhizobium Product 

A 200 g batch (~5683 seeds) of silky lupine seeds was coated in a rotary-drum seed coater (Universal Coating Systems, Independence, OR, USA), with the rotary pan set at 20% of the maximum speed. Seeds were obtained from the Utah Division of Wildlife Resources Great Basin Research Center and Seed Warehouse. Seed testing before the trial showed the seeds had a 66% viability (performed with a tetrazolium chloride test) and 96% purity. The weight of 1000 seeds of silky lupine is equal to 35 g. During coating, the seeds were initially wet with 20 mL of polyvinyl alcohol (PVA) binder (Ashland Inc., Covington, KY, USA) prepared with a 20% solid content and then 25 g of calcium bentonite to create a base layer. This base layer was then covered in 2.5 g of EXCEED^®^ inoculum sandwiched between 2 layers of blended wood flour (160 g), powdered cellulose (30 g), and calcium bentonite (10 g) applied with ~160 mL of PVA binder. No binder was applied with the inoculum to reduce wetting and activating the rhizobium bacteria. The coated seeds were dried at room temperature (~21 °C) in a forced air dryer (Braceworks Automation and Electric, Lloydminster, SK, Canada) for ~30 min and stored in the fridge at 4 °C until planting. 

The performance of the rhizobium-coated seeds was compared against untreated seeds (control). Eight seeds of each treatment were sown separately (5 mm deep) in 7.62 cm^2^ × 10.16 cm deep polycarbonate plant tissue-culture boxes (Plantmedia, Dublin, OR, USA) filled with a sterilized 4:1 mixture of a coarse porous medium of baked clay (Turface^®^ Athletics MVP, Profile Products LLC, Buffalo Grove, IL, USA) and ground vermiculite. Each box had four 3 mm holes and one 8 mm hole drilled in the bottom to allow for water drainage. To maintain moisture in the box and prevent contamination, we inverted an additional plant tissue-culture box over the top of the boxes that contained the plants and growing medium. This “lid” had an 8 mm hole drilled on the top, which was covered with filter tape to allow for air circulation, while also maintaining sterility. 

On 8 April 2021, the plant tissue-culture boxes were placed in a walk-in growth chamber (Environmental Growth Chambers, Chagrin Falls, OH, USA) and held at a constant temperature of 15 °C. This incubation temperature was chosen to mimic spring conditions, when seedlings are actively growing during the early seedling stages in the Great Basin, USA, sagebrush steppe. Lights in the chamber provided a 12 h photoperiod, with a maximum photosynthetically active radiation flux density of approximately 780 µmol m^−2^s^−1^, at plant height. Boxes were arranged according to treatment to decrease the likelihood of contamination and randomized weekly. Plants were watered as needed exclusively with a nitrogen-free watering solution (Table 2) for 10 weeks.

After ten weeks, plant height was measured from the soil surface, and the seedlings were harvested. Biomass was harvested by washing the growing medium from the roots and then drying it in an oven at 105 °C for four days. During harvest, the presence of nodules was noted for each treatment. To have enough biomass for measuring nitrogen content, replicate samples were combined into one. Total nitrogen was analyzed via combustion (Vario EL Cube, Elementar, Langenselbold, Germany).

The effect of the commercial rhizobium inoculum on emergence and plant height was analyzed using generalized linear mixed-effect models [54]. Emergence and plant-height data were fit to a Beta and normal distribution, respectively. Student’s *t*-test was used to test the level of difference between treatments. The significance level was set at *p* < 0.05 for all comparisons. 

### 4.3. Inoculation Trial: Evaluation of Indigenous Rhizobia Strains on Root Nodulation and Plant Growth When Applied as a Liquid Culture on Pre-Germinated Seeds

We evaluated six different inoculum treatments: control, Exceed^®^ H Type Inoculant for Lupine (commercial peat inoculum), a liquid culture of the rhizobia in the Exceed^®^ H Type Inoculant for Lupine (commercial liquid inoculum), and liquid cultures from each of the three native strain collections (Rio Tinto, Strawberry, and Draper). Due to our inability to obtain more samples of silky lupine, we conducted the research on silvery lupine. Again, seeds were obtained from the Utah Division of Wildlife Resources Great Basin Research Center and Seed Warehouse. Seed testing before the trial showed that the seeds had a 59% viability and 96% purity. The weight of 1000 seeds of silvery lupine are equal to 25 g. Before the application of the inoculum, seeds were pre-germinated in 15 cm diameter Petri dishes with two layers of blue blotter paper (Anchor Paper Co., St. Paul, MN, USA). On 28 April 2022, germinates were transplanted into plant tissue-culture boxes prepared as described above, two per box. After 7 d, the seedlings were treated with one of the six inoculum treatments. For the commercial peat inoculum, we applied 1 g of the product per box (0.5 g per plant). Liquid inoculums were diluted to an OD of 0.05 nm and resuspended in ddH_2_O. These solutions were applied directly to the box, using a sterile pipet. Each plant was given 500 µL of inoculated water or ddH_2_O depending on the treatment. The boxes containing the different treatments were arranged in the growth chamber, with six replicates per treatment, under a completely randomized design. Boxes were re-randomized each week. Plants were watered with ddH_2_O for the first two weeks (until seedlings had begun to establish), and for the remainder of the study, a nitrogen-free fertilizer (Table 2) was included with the water. After ten weeks, plants were harvested, and we measured shoot length, above- and belowground biomass, and number of root nodules. Biomass was assessed by washing the growing medium from the roots and then drying it in an oven at 105 °C for two days. Total nitrogen for both shoots and roots was analyzed via combustion (LECO TruSpec CN Determinator, LECO Instruments, St. Joseph, MI, USA).

We analyzed the effect of the rhizobium inoculum treatments on shoot length, above- and belowground biomass, and number of nodules using generalized models with a Poisson distribution (JMP^®^ Pro 16; SAS Institute Inc., Cary, NC, USA). Four plant tissue-culture boxes did not produce plants, and they were not included in the analysis. We performed pairwise comparisons between treatments using the Tukey HSD pairwise comparison test. A significance level of *p* < 0.05 was used for all comparisons. 

### 4.4. Coating Trial: Evaluation of Indigenous Rhizobia Strains When Applied with Different Seed Coatings

Silvery lupine seeds were scarified to weaken the hard seed coat and improve germination uniformity by soaking in 98% sulfuric acid for 4 min, followed by thorough washing with distilled water for 10 min. After scarification, seeds were left to dry under ambient conditions prior to coating for 24 h. Seeds were either coated as in Trial 1 (hereafter referred to as Standard Coating (SC)) or with this same coating, except the wood flour, powdered cellulose, and calcium bentonite were replaced with LifeCube Compost & Soil Builder (TeaLab, Eureka, CA, USA) (hereafter referred to as compost coating (CC)) (Table 3). LifeCube Compost & Soil Builder is composed of a blend of compost, biochar, worm castings, insect frass, biokashi, kelp meal, rock dust, and alfalfa meal. These two different seed-coating treatments were either applied to the seed alone or with one of the four different rhizobia inoculum strains described in the Inoculation Trial: Exceed^®^ H Type Inoculant for Lupine, and indigenous strain collections at Rio Tinto, Strawberry, and Draper (11 treatments total; Table 4). 

The trial was run in the same plant tissue-culture boxes, growing medium, growing conditions (i.e., light, temperature, water, and fertilizer), and arrangement as described in the previous study. Plant tissue-culture boxes contained 16 seeds each. The study was installed on 17 May 2022, with plant tissue-culture boxes arranged in a completely randomized design in the growth chamber, with 11 replicates per treatment. Each box was thinned to the three healthiest plants two weeks after planting. Seedlings that emerged after thinning were removed. Ten weeks after planting, we measured the same metrics as described in the Inoculation Trial, with the addition of ranking the nodule color from 0 to 5. Plants with no nodules were ranked 0, and plants with dark pink nodules were ranked 5. Older nodules that were white, brown, or green were given a value of 1. 

We analyzed the effect of the rhizobium inoculum treatments on seedling emergence, number of nodules, shoot length, and both above- and belowground biomass using generalized regression models (JMP^®^ Pro 16; SAS Institute Inc., Cary, NC, USA). In the models, emergence, number of nodules, and shoot length were fit with beta, Poisson, and log-normal distributions, respectively. The above- and belowground biomass were both fitted with normal distributions. For the above- and belowground biomass, the model included all the treatments. A second analysis was performed with just the treatments that did not contain a rhizobia treatment to assess the effects of the coatings alone. A Tukey HSD pairwise comparison test was used to analyze differences among all treatments. In addition, Dunnett’s multiple comparison test was used to assess differences in treatment means compared to the control. A significance level of *p* < 0.05 was used for all comparisons.

## Figures and Tables

**Table 1:** Batch recipe used to make tryptone yeast (TY) broth from which 250 mL of the solution is removed, mixed with 3 g of bacterial agar, and poured into Petri dishes.

Ingredient	Amount
Autoclaved, ddH_2_O	1 L
Tryptone	5 g
Yeast extract	2.5 g
CaCl_2_·2H_2_O	0.4 g
MgSO_4_·7H_2_O	0.4 g
KOH	300 µL

**Table 2:** Nitrogen-free fertilizer solution; each ingredient was autoclaved prior to use, except for the micronutrients, which were filter sterilized.

INGREDIENT		AMOUNT
AUTOCLAVED, DDH_2_O		500 mL
1M KH_2_PO_4_ [pH~7.0]		1 mL
1M CACL_2_·2H_2_O		0.25 mL
1M MGSO_4_·7H_2_O		0.50 mL
3M KCL		0.50 mL
MICRO (SEE BELOW)		0.50 mL
MICRO	EDTA	230 mg
	FeCl_3_	100 mg
	ZnSO_4_	50 mg
	H_3_BO_3_	5 mg
	CuSO_4_	5 mg
	Na_2_MoO_4_	5 mg
	CoCl_2_	5 mg
	MnSO_4_	50 mg

**Table 3:** Standard coating (SC) and compost coating (CC) recipes applied to 90 g of silvery lupine seed (*Lupinus argenteus* Pursh).

Coating	Ingredient	Amount (g)
SC	Wood flour (system 3)	72
	Powdered cellulose (J Rettenmaier)	13.5
	Calcium Bentonite	4.5
	Peat moss (Black Gold), blended	27.5
CC	Compost (TeaLab), sieved to #12 Tyler mesh sieve	129.6
	Peat moss (Black Gold)	24.75

**Table 4:** Treatments used in the seed-coating trial consisting of either standard coating (SCs) or compost coating (CC) in conjunction with a commercial inoculum, EXCEED^®^ (Ex), or indigenous inoculum collected from Rio Tinto Kennecott Mine, Herriman, UT (RT); from Draper, UT (Dr); or near Strawberry Reservoir, Heber, UT (St). Treatments were applied to silvery lupine (*Lupinus argenteus* Pursh) seeds.

Treatment	Description
Control	Uncoated seed
SC	Seed coated with no added inoculum
CC	Seed coated with compost and no added inoculum
SC + Ex	SC with EXCEED^®^
SC + RT	SC with the native strain collected from Rio Tinto Kennecott Mine
SC + Dr	SC with the native strain collected near Draper, UT
SC + St	SC with the native strain collected near Strawberry Reservoir, UT
CC + Ex	CC with EXCEED^®^
CC + RT	CC with the native strain collected from Rio Tinto Kennecott Mine
CC + Dr	CC with the native strain collected from Draper, UT
CC + St	CC with the native strain collected near Strawberry Reservoir, UT

## Data Availability

The raw data supporting the conclusions of this article will be made available by the authors upon request.
